# Semisynthetic flavonoid 7-*O*-galloylquercetin activates Nrf2 and induces Nrf2-dependent gene expression in RAW264.7 and Hepa1c1c7 cells

**DOI:** 10.1016/j.cbi.2016.10.015

**Published:** 2016-12-25

**Authors:** Lenka Roubalová, David Biedermann, Barbora Papoušková, Jan Vacek, Marek Kuzma, Vladimír Křen, Jitka Ulrichová, Albena T. Dinkova-Kostova, Jiří Vrba

**Affiliations:** a Department of Medical Chemistry and Biochemistry, Faculty of Medicine and Dentistry, Palacký University, Hněvotínská 3, Olomouc 77515, Czech Republic; b Institute of Molecular and Translational Medicine, Faculty of Medicine and Dentistry, Palacký University, Hněvotínská 3, Olomouc 77515, Czech Republic; c Institute of Microbiology, Laboratory of Biotransformation, Czech Academy of Sciences, Vídeňská 1083, Prague 14220, Czech Republic; d Regional Centre of Advanced Technologies and Materials, Department of Analytical Chemistry, Faculty of Science, Palacký University, 17. listopadu 12, Olomouc 77146, Czech Republic; e Jacqui Wood Cancer Centre, Division of Cancer Research, School of Medicine, University of Dundee, Dundee DD1 9SY, Scotland, UK

**Keywords:** Quercetin, Quercetin-7-gallate, Methyl gallate, Nrf2, Heme oxygenase-1, Metabolism, ARE, antioxidant response element, DMSO, dimethyl sulfoxide, ERK, extracellular signal-regulated kinase, Gapdh, glyceraldehyde-3-phosphate dehydrogenase, GCLC, glutamate-cysteine ligase catalytic subunit, GCLM, glutamate-cysteine ligase modifier subunit, HO-1/Hmox1, heme oxygenase-1, JNK, c-Jun N-terminal kinase, Keap1, Kelch-like ECH-associated protein 1, MAPK, mitogen-activated protein kinase, MTT, 3-(4,5-dimethylthiazol-2-yl)-2,5-diphenyltetrazolium bromide, NAC, *N*-acetyl-l-cysteine, NQO1, NAD(P)H:quinone oxidoreductase 1, Nrf2, NF-E2 p45-related factor 2, p38 MAPK, p38 mitogen-activated protein kinase, ROS, reactive oxygen species

## Abstract

The natural flavonoid quercetin is known to activate the transcription factor Nrf2, which regulates the expression of cytoprotective enzymes such as heme oxygenase-1 (HO-1) and NAD(P)H:quinone oxidoreductase 1 (NQO1). In this study, a novel semisynthetic flavonoid 7-*O*-galloylquercetin (or quercetin-7-gallate, **3**) was prepared by direct galloylation of quercetin, and its effect on the Nrf2 pathway was examined. A luciferase reporter assay showed that 7-*O*-galloylquercetin, like quercetin, significantly activated transcription via the antioxidant response element in a stably transfected human AREc32 reporter cell line. In addition, 7-*O*-galloylquercetin caused the accumulation of Nrf2 and induced the expression of HO-1 at both the mRNA and protein levels in murine macrophage RAW264.7 cells. The induction of HO-1 by 7-*O*-galloylquercetin was significantly suppressed by *N*-acetyl-l-cysteine and SB203580, indicating the involvement of reactive oxygen species and p38 mitogen-activated protein kinase activity, respectively. HPLC/MS analyses also showed that 7-*O*-galloylquercetin was not degalloylated to quercetin, but it was conjugated with glucuronic acid and/or methylated in RAW264.7 cells. Furthermore, 7-*O*-galloylquercetin was found to increase the protein levels of Nrf2 and HO-1, and also the activity of NQO1 in murine hepatoma Hepa1c1c7 cells. Taken together, we conclude that 7-*O*-galloylquercetin increases Nrf2 activity and induces Nrf2-dependent gene expression in RAW264.7 and Hepa1c1c7 cells.

## Highlights

• A novel semisynthetic flavonoid was prepared: 7-*O*-galloylquercetin (**3**).
• **3** activates Nrf2/antioxidant response element in AREc32 reporter cells.
• **3** increases Nrf2 and Nrf2-regulated proteins in RAW264.7 and Hepa1c1c7 cells.
• **3** is not hydrolyzed, but it is glucuronidated and/or methylated in RAW264.7 cells.

## Graphical abstract

**Figure:**
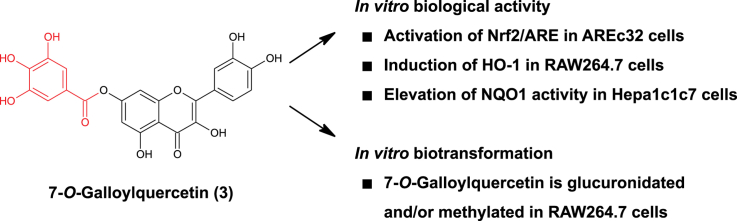

## 1 Introduction

**Fig. 1:**
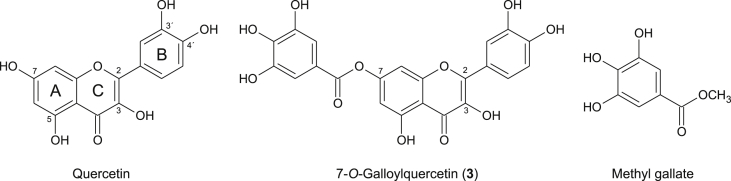
Chemical structures of tested compounds.

Natural flavonoids are a large group of extensively studied plant phenolic compounds. The basic flavonoid structure consists of two benzene rings (A and B) connected by three carbons that form an oxygenated heterocycle (ring C). The flavonoid skeletons such as flavone and flavanone may bear different numbers of hydroxyl groups, modifications of which can yield a multitude of compounds [1]. The flavonol quercetin (3,5,7,3′,4′-pentahydroxyflavone; Fig. 1), arguably the most abundant and most studied bioflavonoid, is predominantly found in plants in the form of its glycosides and methyl ethers, such as rutin (quercetin-3-*O*-rutinoside), isoquercitrin (quercetin-3-*O*-β-D-glucoside) and isorhamnetin (3′-*O*-methylquercetin) [2]. Naturally occurring conjugated forms of quercetin also include those produced by the biotransformation processes in the intestine and liver of animals and humans. The main quercetin conjugates found in human plasma are quercetin-3-*O*-glucuronide, 3′-*O*-methylquercetin-3-*O*-glucuronide and quercetin-3′-*O*-sulfate [3]. In addition, a number of quercetin derivatives have been prepared *in vitro* using chemical or enzymatic procedures. Some derivatives have been designed mainly with the aim of improving the physico-chemical and biological properties of quercetin, which is, despite its beneficial (e.g. anti-inflammatory) effects, unsuitable for medical applications due to low solubility and low bioavailability [4]. Moreover, methods for the preparation of quercetin metabolites have also been developed [5], [6]. The quercetin derivatives synthesized to date include, for instance, esters of quercetin and of various aliphatic [7], [8] and aromatic carboxylic acids [9], [10], [11], quercetin-amino acid conjugates with amino acids attached to quercetin via a carbamate linkage [12], esters of sulfuric acid (i.e. quercetin sulfates) [13], [14], glycosides [15], glucuronides [5], methyl ethers [16], etc.

One of the mechanisms mediating the beneficial action of quercetin is the activation of the transcription factor NF-E2 p45-related factor 2 (Nrf2; also called NFE2L2) [17], [18]. Nrf2 regulates the expression of genes encoding various cytoprotective enzymes such as heme oxygenase-1 (HO-1), NAD(P)H:quinone oxidoreductase 1 (NQO1), and glutamate-cysteine ligase catalytic (GCLC) and modifier subunits (GCLM) [19]. Being inspired by the fact that the Nrf2 activation by catechins (flavanols) positively depends on the presence of a galloyl moiety in the molecule [20], we have previously prepared galloyl esters of quercetin and of its natural derivative, the flavanonol taxifolin (2,3-dihydroquercetin), and examined their effect on the Nrf2 pathway in murine macrophage RAW264.7 cells. We have found that 3-*O*-galloylquercetin, in contrast to quercetin, does not activate the Nrf2 pathway. On the other hand, we have demonstrated the activation of Nrf2 in cells treated with 7-*O*-galloyltaxifolin, while mere taxifolin was inactive. The same study also found that 7-*O*-galloyltaxifolin was readily oxidized to 7-*O*-galloylquercetin (Fig. 1) in RAW264.7 cells [11]. Since it was unclear which of these two molecules was responsible for the biological response, we decided to prepare 7-*O*-galloylquercetin and to investigate its effect on the Nrf2 pathway *in vitro*.

## 2 Experimental

### 2.1 General

NMR analyses were performed using a Bruker Avance III 600 MHz spectrometer (Bruker Biospin, Rheinstetten, Germany) (600.23 MHz for ^1^H, 150.93 MHz for ^13^C) with samples dissolved in DMSO-*d*_6_ (99.8% atom D; VWR International, Leuven, Belgium). NMR spectra were referenced by the residual signal of the solvent (*δ*_H_ 2.500 ppm, *δ*_C_ 39.60 ppm). NMR experiments, including ^1^H NMR, ^13^C NMR, COSY, ^1^H-^13^C HSQC and ^1^H-^13^C HMBC, were performed using the software Topspin3 (Bruker Biospin). The 1D spectra, i.e. ^1^H NMR and ^13^C NMR spectra, were zero-filled to four-fold data points prior to the Fourier transformation. Moreover, ^1^H NMR data were multiplied by the two-parameter double-exponential Lorentz-Gauss function to improve resolution, and line broadening (1 Hz) was applied to the ^13^C NMR data to improve the signal-to-noise ratio. Chemical shifts are listed in δ-scale and coupling constants in Hz. The digital resolution enabled us to declare values to three (*δ*_H_) or two (*δ*_C_) decimal places. ESI-MS spectra were measured with a Micromass LC-MS Platform in MeOH with the addition of HCO_2_H; HRMS spectra (ESI, APCI, FAB) were measured with an LTQ Orbitrap XL instrument (Thermo Fisher Scientific, Waltham, MA, USA) or with a ZAB-EQ instrument (VG Analytical, Manchester, UK).

### 2.2 Synthesis of 7-*O*-galloylquercetin (**3**)

3,4,5-Tri-*O*-benzylgallic acid (1 g, 2.5 mmol) was suspended in dry dichloromethane (15 mL) under an argon atmosphere. Oxalyl chloride (4 mL, 46.6 mmol) was added to the solution, and dry dimethylformamide (2 mL, 26.0 mmol) was added to the resulting mixture dropwise under stirring. After another 4 h of stirring, the reaction mixture was diluted with dry toluene (5 mL) and evaporated to dryness *in vacuo*. The resulting white crude 3,4,5-tri-*O*-benzylgalloyl chloride was dissolved in dry pyridine (15 mL).

Quercetin (0.5 g, 1.65 mmol), previously dried by coevaporation with dry toluene (90 °C, 3 × 10 mL) *in vacuo*, was dissolved in dry pyridine (5 mL). This solution was added to the cooled (−60 °C) solution of 3,4,5-tri-*O*-benzylgalloyl chloride under an argon atmosphere and the reaction mixture was stirred overnight under argon to slowly reach room temperature. Solvents were evaporated and then coevaporated with toluene *in vacuo* to remove traces of pyridine. The resulting solid was mixed with chloroform and water; the organic phase was separated, washed with water, dried with Na_2_SO_4_ and evaporated to dryness. Flash chromatography on Silicagel 60 (CHCl_3_/toluene/acetone/HCO_2_H, 85:5:5:1) yielded 7-*O*-(3′,4′,5′-tri-*O*-benzylgalloyl)quercetin (**1**; 260 mg, 27%) and 3-*O*-(3′,4′,5′-tri-*O*-benzylgalloyl)quercetin (**2**; 180 mg, 19%), which was not used in further experiments.

**1** (260 mg) was dissolved in EtOAc (20 mL), and then Pd/C (150 mg, 10% Pd) was added and stirred under H_2_ for 3 h (20 °C). Palladium catalyst was then removed by filtration through celite, which was washed with acetone, and the solvent was evaporated. The product was purified by gel filtration (Sephadex LH-20, MeOH) yielding 7-*O*-galloylquercetin (**3**; 180 mg, 89%). The purity (HPLC-PDA) was 99.6%, HRMS (ESI) *m/z* [M − H]^-^ calcd for C_22_H_13_O_11_ 453.04524, found 453.04553 (for NMR data see Table S1 and Figs. S2–S6, for HPLC see Fig. S7, for MS and HRMS see Figs. S8 and S9 in Supplementary Information).

### 2.3 Reagents for biological testing

Quercetin, gallic acid methyl ester, hemin, sulforaphane, *N*-acetyl-l-cysteine, PD98059 (2-(2-amino-3-methoxyphenyl)-4*H*-1-benzopyran-4-one), SB203580 (4-(4-fluorophenyl)-2-(4-methylsulfinylphenyl)-5-(4-pyridyl)-1*H*-imidazole), SP600125 (1,9-pyrazoloanthrone), dimethyl sulfoxide (DMSO) and solvents for HPLC were purchased from Sigma-Aldrich.

### 2.4 Cell cultures and treatments

The stable human mammary AREc32 reporter cell line [21] was cultured in Dulbecco's modified Eagle's medium (DMEM; No. 41966, Gibco, Grand Island, NY, USA) supplemented with 2 mM glutamine and 10% fetal bovine serum (FBS). The murine macrophage RAW264.7 cell line (No. 91062702, ECACC, Salisbury, UK) was cultured in DMEM (D5796, Sigma) supplemented with 100 U/mL penicillin, 100 μg/mL streptomycin and 10% FBS. The murine hepatoma Hepa1c1c7 cell line (No. 95090613, ECACC) was cultured in Minimum essential medium α (M0894, Sigma) supplemented with 2.2 g/L NaHCO_3_ and 10% heat- and charcoal-treated FBS. Cells were maintained at 37 °C in a humidified atmosphere containing 5% CO_2_. For experiments, cells were seeded into multiwell plates in serum-containing medium. Experiments on AREc32 and Hepa1c1c7 cells were performed after 24 h of stabilization in fresh serum-containing medium. For RAW264.7 cells, the culture medium was replaced with serum-free medium after 8 h of stabilization, and following overnight incubation the experiments were performed in fresh serum-free conditions. Cells were treated with the tested compounds (in 0.1% (*v/v*) DMSO) and negative controls were treated with 0.1% DMSO alone.

### 2.5 Cell viability assay

RAW264.7 cells (1.7 × 10^5^ cells/well in a 24-well plate) and Hepa1c1c7 cells (1 × 10^4^ cells/well in a 96-well plate) were treated for 6 or 48 h, respectively, with 0.1% DMSO (control), 1.5% (*v/v*) Triton X-100 (positive control) or with the tested compounds. After treatment, the cell viability was determined using the MTT reduction assay. Cells were washed with phosphate-buffered saline (PBS) and incubated for 2 h at 37 °C in fresh serum-free medium containing 0.5 mg/mL 3-(4,5-dimethylthiazol-2-yl)-2,5-diphenyltetrazolium bromide (MTT). After this, the medium was removed and formazan produced by active mitochondria was dissolved in DMSO. The absorbance at 540 nm was measured on a spectrophotometric plate reader and used to calculate relative cell viability, where cells treated with DMSO alone represented 100% viability.

### 2.6 Gene reporter assay

AREc32 cells were seeded into a 96-well plate at 1 × 10^4^ cells/well. On the next day, cells were exposed for 24 h to the tested compounds. Afterwards, the plate was frozen at −20 °C for 24 h and then the luciferase activity was measured on a GloMax-Multi+ microplate luminometer (Promega, Madison, WI, USA) using the Bright-Glo Luciferase Assay System (Promega). The luminescence values were normalized to the protein content of the cells and used for calculation of fold changes versus the control.

### 2.7 Reverse transcription and quantitative real-time PCR

After the treatment of RAW264.7 cells (8 × 10^5^ cells/well in a 6-well plate), total RNA was extracted using TRI Reagent Solution (Applied Biosystems, Foster City, CA, USA). RNA samples (2 μg) were reverse transcribed using a High-Capacity cDNA Reverse Transcription Kit (Applied Biosystems) and real-time PCR was performed in a LightCycler 480 II system (Roche Diagnostics, Mannheim, Germany) using TaqMan Universal PCR Master Mix and TaqMan Gene Expression Assays, consisting of specific primers and FAM dye-labeled TaqMan minor groove binder probes (Applied Biosystems). The assay ID was Mm00516005_m1 for Hmox1 and Mm99999915_g1 for Gapdh. Amplification conditions were 50 °C for 2 min, 95 °C for 10 min, followed by 40 cycles at 95 °C for 15 s and 60 °C for 1 min. Crossing point values, equivalent to *C*_T_, were determined using second derivative maximum analysis. Relative changes in gene expression were calculated by the comparative *C*_T_ method using the $2^{-\Delta \Delta C_{\text{T}}}$ equation with results normalized to Gapdh mRNA levels.

### 2.8 Western blot analysis

After the treatment of RAW264.7 cells (8 × 10^5^ cells/well in a 6-well plate) or Hepa1c1c7 cells (4 × 10^5^ cells/well in a 6-well plate), total cellular extracts were prepared as described previously [11]. Aliquots containing an equal amount of protein were subjected to electrophoresis through 4–12% sodium dodecyl sulfate-polyacrylamide gel, proteins were transferred to polyvinylidene difluoride membrane by electroblotting, and the membranes were probed with appropriate primary antibodies. Rabbit polyclonal heme oxygenase-1 (sc-10789), rabbit polyclonal Nrf2 (sc-722), goat polyclonal Keap1 (sc-15246) and goat polyclonal actin (sc-1616) antibodies were obtained from Santa Cruz Biotechnology (Santa Cruz, CA, USA). Antibodies against NQO1, GCLC and GCLM were kindly provided by Dr. John D. Hayes (University of Dundee, Dundee, UK). Primary antibodies were visualized with rabbit anti-goat or goat anti-rabbit horseradish peroxidase-conjugated secondary antibodies using a chemiluminescent reaction.

### 2.9 HPLC/MS analysis of biotransformation products

RAW264.7 cells (8 × 10^5^ cells/well in a 6-well plate) were incubated for 0–6 h with 15 μM 7-*O*-galloylquercetin. After incubation, cells were scraped from the plates, collected by gentle centrifugation, washed twice with PBS, resuspended in 0.4 mL of methanol containing 5% (*v/v*) acetic acid, and sonicated 10 times at 50% amplitude with a cycle set at 0.5 s using an Ultrasonic Processor UP200s equipped with a Sonotrode Microtip S2 sonicator probe (Hielscher, Teltow, Germany). Afterwards, the cell lysates were centrifuged for 2 min at 14 000× *g* at room temperature and the supernatants were analyzed by HPLC/MS. Aliquots of culture medium were diluted (1:1, *v*/*v*) in methanol containing 5% (*v/v*) acetic acid, centrifuged for 2 min at 14 000× *g* and the supernatants were analyzed by HPLC/MS. The chromatographic separation was performed in an Agilent Zorbax Eclipse XDB-phenyl column (150 mm × 2.1 mm i.d., 5 μm; Agilent Technologies, CA, USA) using an Acquity UPLC system (Waters, Milford, MA, USA) equipped with a binary solvent manager, sample manager, column manager and photodiode array detector. A Waters QqTof Premier Mass Spectrometer (Waters, Manchester, UK) was connected to the UPLC system via an electrospray ionization (ESI) interface. Acquiring data enabled the collection of intact precursor ions as well as fragment ion information in an unbiased manner. Post-acquisition processing of the data was performed using Metabolynx V4.1 software (Waters, Milford, MA, USA). For more details, see Ref. [22].

### 2.10 NQO1 activity assay

After the treatment of Hepa1c1c7 cells (1 × 10^4^ cells/well in a 96-well plate), the activity of NQO1 was determined spectrophotometrically as described previously [23]. Cells were washed four times with PBS and lysed with 75 μL of digitonin solution (0.8 g/L digitonin, 2 mM EDTA, pH 7.8) by shaking on an orbital shaker for 20 min at room temperature. One part of the cell lysate (20 μL) was used to determine the protein content. The remaining lysate (55 μL) was mixed with 200 μL of 0.5 M Tris-Cl buffer containing 10% bovine serum albumin, 1.5% Tween-20, 7.5 mM FAD, 150 mM glucose-6-phosphate, 2 U/mL glucose-6-phosphate dehydrogenase (Roche), 50 mM NADP^+^, 25 mM menadione and 0.7 mM MTT. The mixture was incubated for 5 min at room temperature and the reaction was stopped with 50 μL of dicumarol solution (0.3 mM dicumarol, 5 mM potassium phosphate, 0.5% DMSO). The absorbance of the reduced MTT corresponding to the activity of NQO1 was measured at 610 nm on a spectrophotometric plate reader. The absorbance values were normalized to the protein content of the cells and used for the calculation of fold changes versus the control.

### 2.11 Statistical analysis

Results were expressed as means ± standard deviation (SD). The differences in mean values were analyzed by one-way ANOVA with Tukey's post hoc test. A *p* value of less than 0.05 was considered to be statistically significant.

## 3 Results and discussion

### 3.1 Synthesis of 7-*O*-galloylquercetin (**3**)

**Scheme 1:**
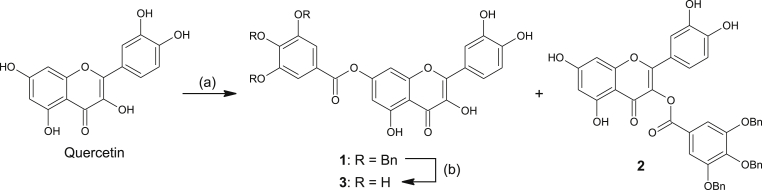
Synthesis of 7-*O*-galloylquercetin (**3**). Reagents and conditions: (a) 3,4,5-tri-*O*-benzylgalloyl chloride (1.5 equiv), pyridine, –60 °C, 1 h, 27%; (b) H_2_–Pd/C, EtOAc, room temp, 3 h, 89%.

The synthesis of **3** was performed by direct galloylation of quercetin with 3,4,5-tri-*O*-benzylgalloyl chloride in pyridine (Scheme 1). 3,4,5-Tri-*O*-benzylgalloyl chloride in pyridine was prepared by treating tribenzylgallic acid with oxalyl chloride solution in CH_2_Cl_2_ and a catalytic amount of dimethylformamide immediately before use. The reaction yielded two regioisomers **1** and **2** that were separated by flash chromatography. Benzyl-protected intermediate **1** was then deprotected by catalytic hydrogenolysis catalyzed with Pd/C, giving the final galloyl ester **3**.

### 3.2 Effect of 7-*O*-galloylquercetin (**3**) on cell viability

**Fig. 2:**
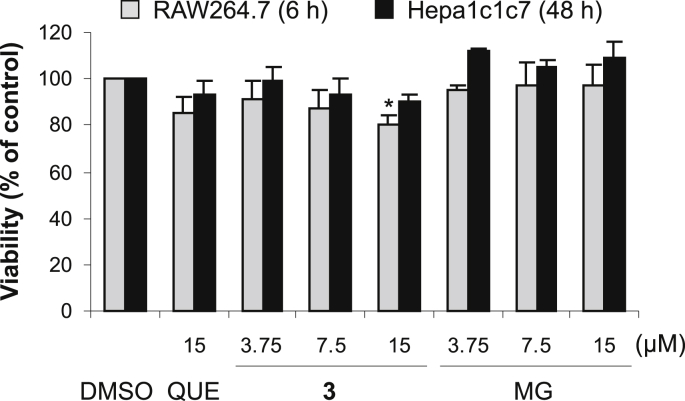
Effect of tested compounds on viability of RAW264.7 and Hepa1c1c7 cells. RAW264.7 and Hepa1c1c7 cells were treated for 6 or 48 h (as indicated) with 0.1% DMSO (control), 15 μM quercetin (QUE) or with 3.75–15 μM 7-*O*-galloylquercetin (**3**) or methyl gallate (MG). The cell viability was determined by the MTT reduction assay. Data are means ± SD of three experiments. *p < 0.05, significantly decreased versus control.

The aim of the biological part of the study was to investigate whether **3** activates the Nrf2 pathway. For that purpose, we used three cell models including murine macrophage RAW264.7 cells, murine hepatoma Hepa1c1c7 cells, and stably transfected AREc32 reporter cells derived from human breast cancer MCF7 cells [21]. To compare the effect of **3** with those of its structural components, we also included quercetin and gallic acid methyl ester (methyl gallate) in our experiments (Fig. 1). Methyl gallate was chosen instead of free gallic acid, since the acid was previously shown to have only a weak stimulating effect on the Nrf2 pathway [11]. In this study, we tested both **3** and its structural components at concentrations of up to 15 μM. At these concentrations, the tested compounds had a weak or negligible effect on the viability of cells used. We found that **3** only decreased cell viability below 90% in RAW264.7 cells, and this effect was comparable to that of quercetin. As shown by the MTT reduction assay, the viability of RAW264.7 cells treated for 6 h with 15 μM **3** or quercetin was 80% and 86%, respectively. The effect of the tested compounds on the viability of RAW264.7 and Hepa1c1c7 cells after 6 and 48 h of incubation, respectively, is shown in Fig. 2.

### 3.3 Activation of Nrf2 by 7-*O*-galloylquercetin (**3**) in AREc32 reporter cells

**Fig. 3:**
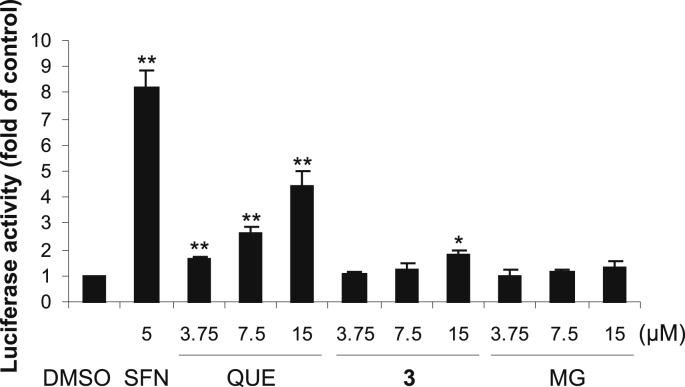
7-*O*-Galloylquercetin (**3**) induces ARE-driven gene expression. AREc32 reporter cells were treated for 24 h with 0.1% DMSO (control), 5 μM sulforaphane (SFN; positive control) or with 3.75–15 μM **3**, quercetin (QUE) or methyl gallate (MG). After treatment, luciferase reporter activity was determined luminometrically and normalized to protein content. Data are means ± SD of three experiments. *p < 0.05; **p < 0.01, significantly increased versus control.

To evaluate the effect on the Nrf2 pathway, we first examined whether compound **3** and its structural components could induce the transcriptional activity of Nrf2 in the stably transfected human AREc32 reporter cell line. AREc32 cells contain a luciferase reporter gene controlled by eight copies of the antioxidant response element (ARE), through which Nrf2 activates the expression of its target genes [21]. The treatment of AREc32 cells for 24 h with 5 μM sulforaphane, used as a positive control [21], resulted in an 8.2-fold increase in luciferase activity compared to the control (Fig. 3). A significant increase in luciferase activity was also induced by **3** and quercetin, with the latter compound being more effective. In contrast, a mild or negligible elevation in luciferase activity was detected after cell exposure to methyl gallate. After 24 h of incubation, the activity of luciferase reached 4.4-fold, 1.8-fold and 1.3-fold in cells treated with 15 μM quercetin, **3** and methyl gallate, respectively (Fig. 3). These results demonstrated that **3** activates the Nrf2-dependent transcription, albeit with a lower potency than quercetin.

### 3.4 Induction of HO-1 by 7-*O*-galloylquercetin (**3**) in RAW264.7 cells

**Fig. 4:**
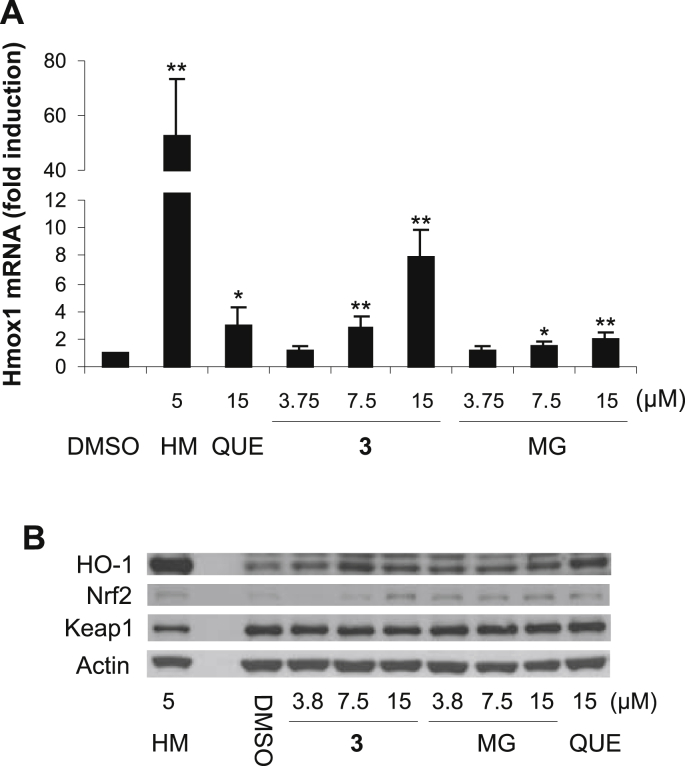
7-*O*-Galloylquercetin (**3**) induces HO-1 expression and Nrf2 accumulation in RAW264.7 cells. Cells were treated for 6 h with 0.1% DMSO (control), 5 μM hemin (HM; positive control), 15 μM quercetin (QUE) or with 3.75–15 μM **3** or methyl gallate (MG). (A) After treatment, relative changes in Hmox1 mRNA levels were determined by quantitative real-time PCR with results normalized to Gapdh mRNA. Data are means ± SD of five experiments. *p < 0.05; **p < 0.01, significantly increased versus control. (B) After treatment, protein levels of HO-1, Nrf2, Keap1 and actin in the whole cell lysates (20 μg/lane) were analyzed by Western blotting. Representative Western blots are shown.

The study further compared the effect of **3** and its structural components on the expression of HO-1 in RAW264.7 cells. HO-1, encoded by the *Hmox1* gene, is an inducible form of heme oxygenase, an enzyme that degrades heme to Fe^2+^, the antioxidant biliverdin and anti-inflammatory agent carbon monoxide [24]. As shown by quantitative real-time PCR, the treatment of RAW264.7 cells for 6 h with 5 μM hemin, a positive control, significantly increased the level of Hmox1 mRNA to 53-fold when normalized to Gapdh mRNA (Fig. 4A). After 6 h of incubation, **3** was found to induce the expression of the *Hmox1* gene in RAW264.7 cells in a dose-dependent manner, with a significant increase in Hmox1 mRNA detected at concentrations from 7.5 μM. The levels of Hmox1 mRNA were also elevated in cells treated with quercetin and methyl gallate, but their effect was weaker than that of **3**. At the concentration of 15 μM, Hmox1 mRNA levels induced by **3**, quercetin and methyl gallate reached 7.9-fold, 3.0-fold and 2.0-fold, respectively, compared to the control (Fig. 4A). Western blot analysis showed that the induction of *Hmox1* gene expression by **3** was also associated with the upregulation of the HO-1 protein. After 6 h of cell exposure to **3**, the highest level of HO-1 was found at the concentration of 7.5 μM. We also found that quercetin (15 μM) upregulated HO-1 to a level comparable to that induced by **3**, whereas methyl gallate produced lower levels of HO-1 (Fig. 4B). It should be mentioned that the induction of HO-1 in RAW264.7 cells by quercetin was reported previously [11], [25] and the ability of methyl gallate to upregulate HO-1 was demonstrated in mouse mesangial cells [26].

The expression of the *Hmox1* gene is regulated by Nrf2. Under unstressed conditions, Nrf2 is targeted for proteasomal degradation through its interaction with the repressor protein Keap1 (Kelch-like ECH-associated protein 1). Conversely, stress conditions may impair the interaction between Nrf2 and Keap1 via the phosphorylation of Nrf2 or through the oxidation or covalent modification of Keap1, which in turn undergoes proteasomal degradation. The disruption of the Keap1–Nrf2 interaction results in the accumulation of Nrf2 and its translocation to the nucleus, where it binds to the DNA at the ARE and triggers the expression of Nrf2 target genes [27], [28]. Accordingly, the Western blot analysis showed that 5 μM hemin increased the level of Nrf2 and concurrently decreased the level of Keap1 in RAW264.7 cells after 6 h of incubation (Fig. 4B). We found that **3** at a concentration of 15 μM also upregulated Nrf2, but the level of Keap1 remained unchanged after 6 h of exposure. Similarly, quercetin and methyl gallate also increased Nrf2 without affecting Keap1 levels (Fig. 4B). The accumulation of Nrf2 together with the induction of HO-1 indicates that **3**, like quercetin, may activate the Nrf2 pathway in RAW264.7 cells.

### 3.5 Induction of HO-1 by 7-*O*-galloylquercetin (**3**) involves ROS and p38 MAPK activity

**Fig. 5:**
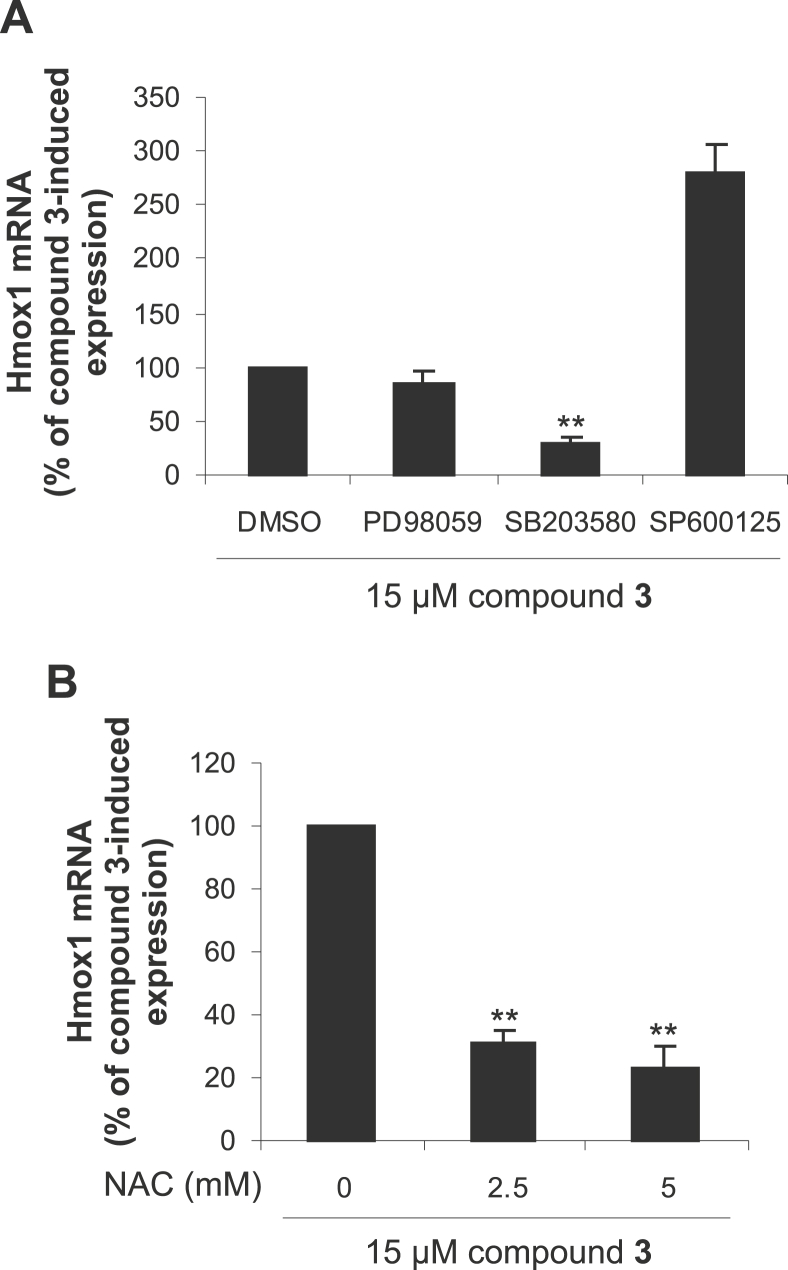
Effect of MAPK inhibitors and *N*-acetyl-l-cysteine (NAC) on 7-*O*-galloylquercetin (compound **3**)-induced *Hmox1* gene expression in RAW264.7 cells. (A) Cells were pretreated for 30 min with 0.1% DMSO (control), 15 μM PD98059, 15 μM SB203580 or 30 μM SP600125 and then incubated in the absence or presence of 15 μM **3** for an additional 6 h. (B) Cells were pretreated for 30 min with 2.5 or 5 mM NAC and then incubated in the absence or presence of 15 μM **3** for an additional 6 h. The levels of Hmox1 mRNA were determined by quantitative real-time PCR and normalized to Gapdh mRNA. Results are expressed as the percentage of compound **3**-induced Hmox1 mRNA expression. Data are means ± SD of three experiments. **p < 0.01, significantly decreased versus **3** in the absence of MAPK inhibitors or NAC.

As described above, the accumulation of Nrf2 in RAW264.7 cells by compound **3** was not associated with a downregulation of Keap1. Since the activation of Nrf2 may also be mediated through its phosphorylation [28], we investigated, using pharmacological inhibitors, whether mitogen-activated protein kinases (MAPKs) including p38 MAPKs, extracellular signal-regulated kinases (ERKs), and c-Jun N-terminal kinases (JNKs) could play a role in the cell response to **3**. We found that the increase in Hmox1 mRNA induced by **3** was reduced significantly by the p38 MAPK inhibitor SB203580, and nonsignificantly by the ERK inhibitor PD98059 (Fig. 5A). Pretreatment of RAW264.7 cells for 30 min with 15 μM SB203580 or 15 μM PD98059 decreased the induction of Hmox1 mRNA by 6 h of exposure to 15 μM **3** by 71% and 15%, respectively. On the other hand, cell pretreatment with 30 μM SP600125, a JNK inhibitor, potentiated the effect of **3** on Hmox1 mRNA expression by 181% under the same experimental conditions (Fig. 5A).

The MAPK pathways may be activated by various stimuli including reactive oxygen species (ROS) [29]. As shown in Fig. 5B, the elevation in Hmox1 mRNA induced in RAW264.7 cells by **3** was significantly suppressed by *N*-acetyl-l-cysteine (NAC), a nonspecific ROS scavenger. For instance, 30 min of the cell pretreatment with 2.5 mM NAC decreased the induction of Hmox1 mRNA by 6 h of exposure to 15 μM **3** by 69% (Fig. 5B). These results suggest that the activation of Nrf2 by **3** is mediated through the enhanced production of ROS and activation of p38 MAPKs. As shown in our previous study [11], both of these events were also involved in HO-1 induction in RAW264.7 cells exposed to 7-*O*-galloyltaxifolin, which only differs from **3** in the absence of a 2,3-double bond. Taking into account the oxidation of 7-*O*-galloyltaxifolin to **3** [11] and essentially the same mechanism of HO-1 induction found for both compounds, it may be supposed that compound **3** was responsible for the biological effect of 7-*O*-galloyltaxifolin in RAW264.7 cells [11].

### 3.6 Uptake and biotransformation of 7-*O*-galloylquercetin (**3**) by RAW264.7 cells

**Fig. 6:**
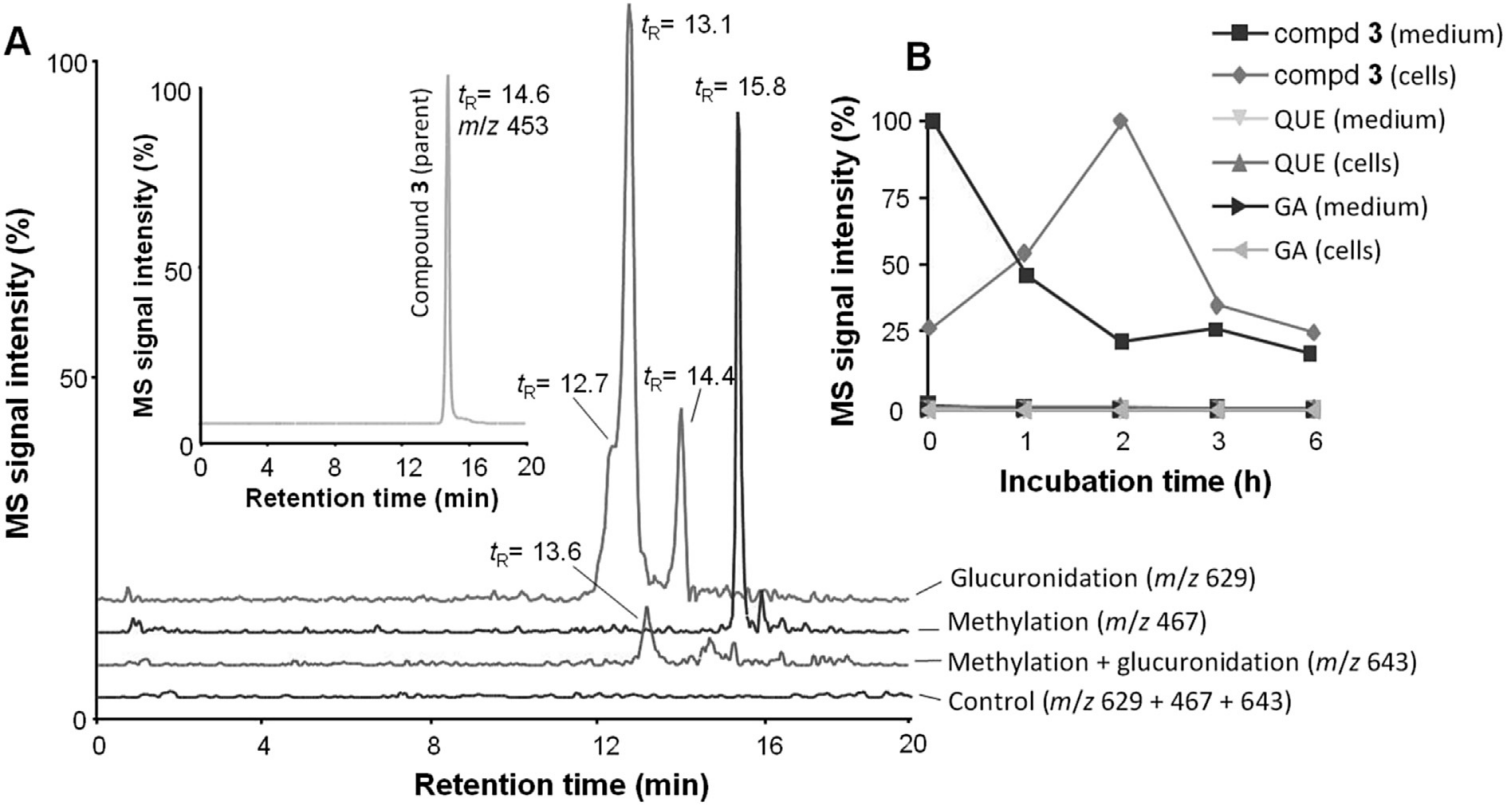
Biotransformation and uptake of 7-*O*-galloylquercetin (**3**) by RAW264.7 cells. Cells were treated for 0–6 h with 15 μM **3** or 0.1% DMSO (control), and cell extracts and culture medium were analyzed by HPLC with negative ESI-MS detection. (A) HPLC/MS chromatograms of **3** (inset) and of its metabolites found in cells after 2 h of treatment. (B) Time course of distribution of **3**, quercetin (QUE) and gallic acid (GA) in cells and medium. Data are means ± SD of three experiments.

**Fig. 7:**
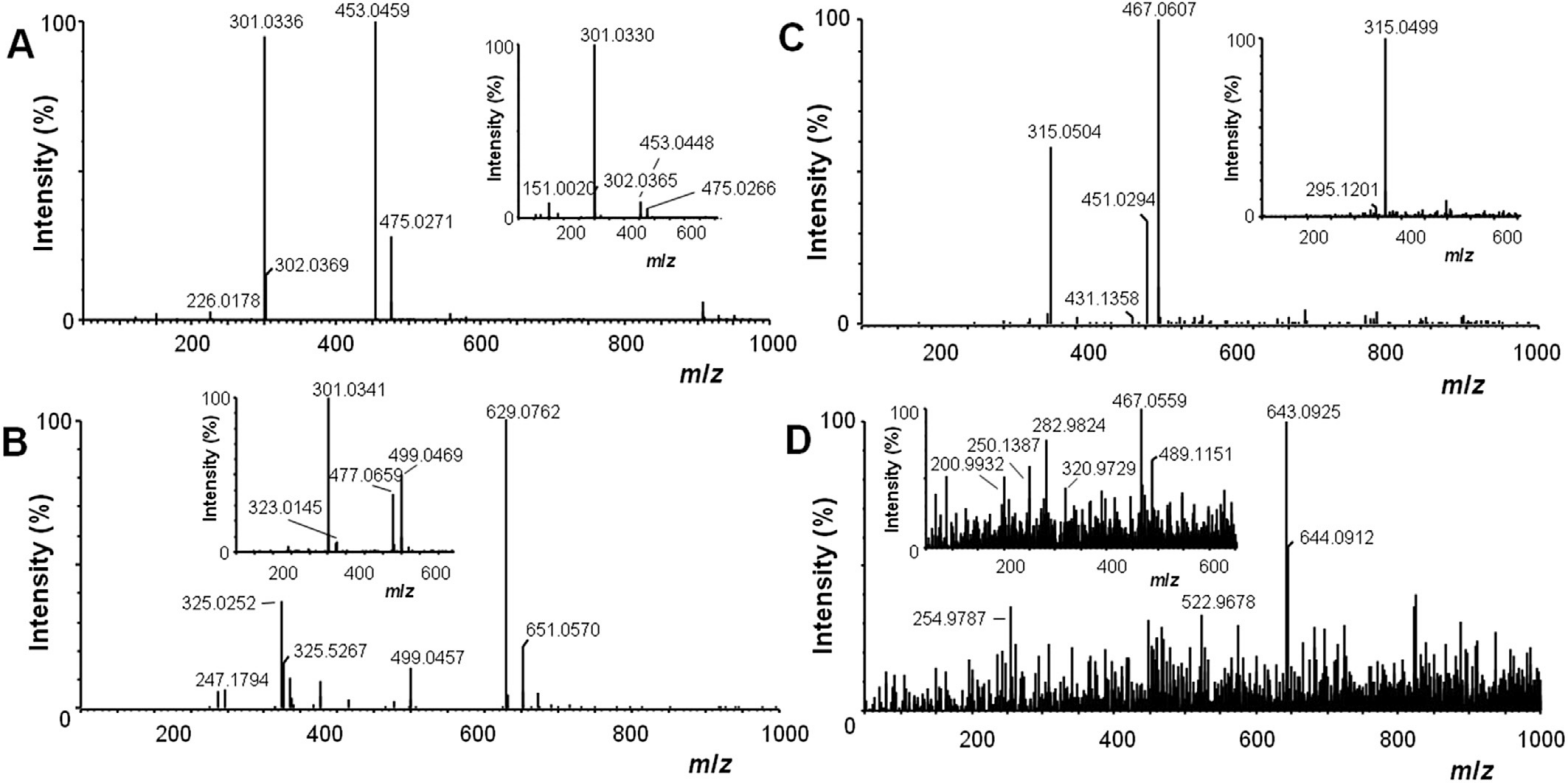
Full and fragmentation (insets) MS spectra of 7-*O*-galloylquercetin (**3**) and of its metabolites. The MS spectra were obtained from the chromatographic peaks shown in Fig. 6. (A) Parent compound **3**, *m/z* 453, (B) glucuronide of **3**, *t*_R_ 13.1 min, *m/z* 629 (C) methylated **3**, *m/z* 467, (D) methylated and glucuronidated **3**, *m/z* 643.

The study also investigated the metabolic fate of **3** in RAW264.7 cells. The cells were incubated for up to 6 h with 15 μM **3**, and the cells and culture medium were separately analyzed using the previously developed high-performance liquid chromatography-mass spectrometry (HPLC/MS) method in Ref. [22]. As recorded with an ESI-MS detector operating in negative mode, compound **3** gave a well-resolved chromatographic peak at a *t*_R_ of 14.6 min (Fig. 6A). We found that during the incubation period, the concentration of **3** in the culture medium evidently decreased. In contrast, the concentration of **3** in cell extracts increased at the beginning of the incubation, reached a maximum at 2 h and then a decline in the concentration occurred (Fig. 6B). These findings indicated that **3** was absorbed by RAW264.7 cells and that the galloyl ester underwent some kind of metabolic transformation and/or degradation. Analyses showed that compound **3** was stable in terms of possible hydrolytic cleavage, since no significant amounts of quercetin and gallic acid were detected in either the cells or medium (Fig. 6B). Thus, we could rule out the possibility that the biological effects of **3** were mediated by its structural components. Furthermore, we found that **3** was not converted to 7-*O*-galloyltaxifolin. On the other hand, two types of phase II conjugation reactions, i.e. glucuronidation and methylation, appeared to be involved in the transformation of **3** in RAW264.7 cells. Analyses revealed three different glucuronides of the parent compound eluted at *t*_R_ of 12.7, 13.1 and 14.4 min, a methyl derivative eluted at a *t*_R_ of 15.8 min, and also a methylated glucuronide that appeared as a minor metabolite at a *t*_R_ of 13.6 min (Fig. 6A). The full and fragmentation MS spectra of **3** and of its metabolites are shown in Fig. 7. We were not able to identify which hydroxyl groups in the molecule of **3** were conjugated. Nonetheless, some of the conjugations could occur at the same positions which are conjugated in quercetin and gallic acid metabolites such as 3′-*O*-methylquercetin-3-*O*-glucuronide [3] and 4-*O*-methylgallic acid [30]. The metabolic activity of RAW264.7 cells was in accordance with the study showing the methylation of quercetin in this cell line [31]. Moreover, the expression of UDP-glucuronosyltransferases was reported in macrophages [32]. It might be mentioned that we previously showed the formation of glucuronides and methyl derivatives of 3-*O*-galloylquercetin in human hepatocytes [22]. Hence, it may be supposed that the biotransformation pattern of **3** found in RAW264.7 cells can also be expected in other cell types expressing the phase II conjugating enzymes.

### 3.7 Induction of NQO1 activity by 7-*O*-galloylquercetin (**3**) in Hepa1c1c7 cells

**Fig. 8:**
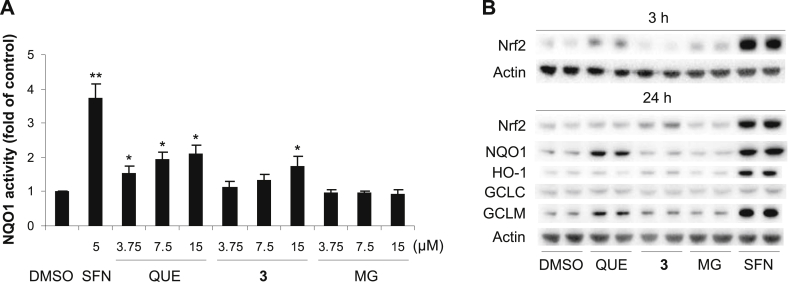
7-*O*-Galloylquercetin (**3**) induces NQO1 activity and Nrf2 accumulation in Hepa1c1c7 cells. (A) Cells were treated for 48 h with 0.1% DMSO (control), 5 μM sulforaphane (SFN; positive control) or with 3.75–15 μM **3**, quercetin (QUE) or methyl gallate (MG). After treatment, the activity of NQO1 was determined using the NQO1 assay. Data are means ± SD of three experiments. *p < 0.05; **p < 0.01, significantly increased versus control. (B) Cells were treated for 3 or 24 h (as indicated) with 0.1% DMSO (control), 5 μM sulforaphane (SFN; positive control) or with 15 μM **3**, quercetin (QUE) or methyl gallate (MG). After treatment, the protein levels of Nrf2, NQO1, HO-1, GCLC, GCLM, and actin in the whole cell lysates (30 μg/lane) were analyzed in duplicate by Western blotting. Representative Western blots are shown.

To evaluate whether **3** could stimulate the Nrf2 pathway in cells other than macrophages, we tested the effect of the compound on murine hepatoma Hepa1c1c7 cells, a well-established model for the identification of possible NQO1 inducers [23]. The NQO1 assay showed that the treatment of Hepa1c1c7 cells for 48 h with 5 μM sulforaphane, a positive control [23], elevated the activity of NQO1 3.7-fold compared to the control (Fig. 8A). The induction of NQO1 activity by sulforaphane was accompanied by an obvious increase in the levels of Nrf2 and Nrf2-regulated proteins, namely, NQO1, HO-1, GCLC and GCLM, as demonstrated by Western blot analysis after 3 and/or 24 h of exposure (Fig. 8B). We found that the activity of NQO1 in Hepa1c1c7 cells was also elevated by **3** and quercetin, but not by methyl gallate. However, **3** significantly increased NQO1 activity only at the highest concentration tested, thus it was a less effective NQO1 inducer than quercetin. In cells treated for 48 h with 15 μM quercetin, **3** and methyl gallate, the activity of NQO1 reached 2.1-fold, 1.8-fold and 0.9-fold, respectively, compared to the control (Fig. 8A). In Hepa1c1c7 cells, **3** (15 μM) was also found to induce a mild increase in the protein levels of Nrf2, HO-1 and GCLM after 24 h of incubation, while the levels of NQO1 and GCLC were unaffected. Quercetin (15 μM) clearly elevated Nrf2 as early as after 3 h of exposure and upregulated NQO1 and GCLM, but not HO-1 and GCLC after 24 h. In contrast, no substantial changes in the levels of Nrf2 and Nrf2-regulated proteins were detected in cells treated with methyl gallate (Fig. 8B). Our results show that the ability of **3** to stimulate the Nrf2 pathway is not restricted to one cell type. The potency of **3** compared to that of quercetin seems, however, to depend on the cell model used.

## 4 Conclusions

The novel semisynthetic flavonoid 7-*O*-galloylquercetin (**3**) has been prepared by the direct galloylation of quercetin. We have demonstrated that **3**, like quercetin, activates the Nrf2 pathway *in vitro*. This is supported by the findings that **3** *i*) activates the antioxidant response element in AREc32 reporter cells, *ii*) induces the accumulation of Nrf2 in RAW264.7 and Hepa1c1c7 cells, *iii*) induces the expression of HO-1 at both the mRNA and protein levels in RAW264.7 cells, and *iv*) increases the activity of NQO1 and protein levels of HO-1 and GCLM in Hepa1c1c7 cells. We have also shown in RAW264.7 cells that the activation of the Nrf2 pathway by **3** involves ROS and p38 MAPK activity, and is not accompanied by the degalloylation of **3** to quercetin. In addition, we have demonstrated that macrophages may contribute to the metabolism of galloylated flavonoids, since **3** was glucuronidated and/or methylated in RAW264.7 cells.

Our results suggest that the galloyl ester **3** is a somewhat less potent activator of Nrf2 than quercetin. Nonetheless, we can speculate that the galloylation also affects other properties relevant to its potential application, such as water-solubility, stability and membrane permeability. This aside, the overall biological activity of **3** may depend, for instance, on the interactions of the galloyl group with ROS [33], transition metal ions [34], [35] and proteins [1]. In conclusion, since Nrf2 plays a crucial role in the maintenance of cellular redox homeostasis, we suggest that further research on compound **3** could be aimed at evaluating its protective potential against oxidative stress induced *in vitro* and/or *in vivo*.
