# Supplementary material for: Semisynthetic flavonoid 7-O-galloylquercetin activates Nrf2 and induces Nrf2-dependent gene expression in RAW264.7 and Hepa1c1c7 cells
Source: Chem Biol Interact. 2016 Dec 25;260:58–66. doi: 10.1016/j.cbi.2016.10.015 (PMC5148792; doi:10.1016/j.cbi.2016.10.015)
Supplement: Supplementary file 1 [file mmc1.pdf]

Supplementary Data

Semisynthetic flavonoid 7-*O*-galloylquercetin activates Nrf2 and induces Nrf2-dependent gene expression in RAW264.7 and Hepa1c1c7 cells

Lenka Roubalová[a,b], David Biedermann[c], Barbora Papoušková[d], Jan Vacek[a], Marek Kuzma[c], Vladimír Křen[c], Jitka Ulrichová[a,b], Albena T. Dinkova-Kostova[e], Jiří Vrba[a,b,]*

[a] *Department of Medical Chemistry and Biochemistry, Faculty of Medicine and Dentistry, Palacký University, Hněvotínská 3, Olomouc 77515, Czech Republic*

[b] *Institute of Molecular and Translational Medicine, Faculty of Medicine and Dentistry, Palacký University, Hněvotínská 3, Olomouc 77515, Czech Republic*

[c] *Institute of Microbiology, Laboratory of Biotransformation, Czech Academy of Sciences, Vídeňská 1083, Prague 14220, Czech Republic*

[d] *Regional Centre of Advanced Technologies and Materials, Department of Analytical Chemistry, Faculty of Science, Palacký University, 17 listopadu 12, Olomouc 77146, Czech Republic*

[e] *Jacqui Wood Cancer Centre, Division of Cancer Research, School of Medicine, University of Dundee, Dundee DD1 9SY, Scotland, UK*

* Corresponding author:

E-mail: vrbambv@seznam.cz

Table of contents **Page**

Table of contents	Page
NMR spectroscopy of 7-*O*-galloylquercetin (**3**)	S3
Figure S1. Structure and numbering system of 7-*O*-galloylquercetin (**3**)	S3
Table S1. ^{1}H and ^{13}C NMR data of 7-*O*-galloylquercetin (**3**)	S4
Figure S2. ^{1}H NMR spectrum of 7-*O*-galloylquercetin (**3**)	S5
Figure S3. ^{13}C NMR spectrum of 7-*O*-galloylquercetin (**3**)	S6
Figure S4. COSY spectrum of 7-*O*-galloylquercetin (**3**)	S7
Figure S5. HSQC spectrum of 7-*O*-galloylquercetin (**3**)	S8
Figure S6. HMBC spectrum of 7-*O*-galloylquercetin (**3**)	S9
Figure S7. HPLC chromatogram of 7-*O*-galloylquercetin (**3**)	S10
Figure S8. MS data of 7-*O*-galloylquercetin (**3**)	S11
Figure S9. HRMS data of 7-*O*-galloylquercetin (**3**)	S12

NMR spectroscopy of 7-*O*-galloylquercetin (3)

Instrument:

Bruker Avance III 600 (600.23 MHz for ^{1}H, 150.94 MHz for ^{13}C, solvent DMSO-d_6, 303.2 K)

Experiments performed:

^{1}H NMR, ^{13}C NMR, gCOSY, ^{1}H-^{13}C proton-edited gHSQC, ^{1}H-^{13}C gHMBC

Referencing:

^{1}H NMR and ^{13}C NMR spectra were referenced using the residual signal of acetone (δ_H 2.500, δ_C 39.60)

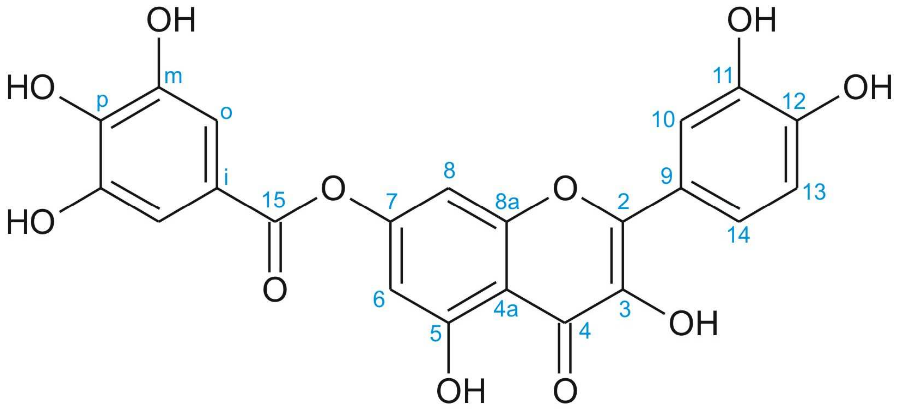

Figure S1. Structure and numbering system of 7-*O*-galloylquercetin (**3**).

Table S1. ^{1}H and ^{13}C NMR data of 7-*O*-galloylquercetin (**3**) (600.23 MHz for ^{1}H, 150.94 MHz for ^{13}C, DMSO-d_6, 303.2 K).

**Atom**	**δ_C [ppm]**	**m**	**δ_H [ppm]**	**n_H**	**m**	**J_{H-H} [Hz]**	**HMBC**
2	148.35	s	-	-	-	-	10, 14, 3-OH
3	136.55	s	-	-	-	-	3-OH*
4	176.32	s	-	-	-	-	8, 3-OH, 5-OH
4a	107.26	s	-	-	-	-	6, 8, 5-OH
5	160.14	s	-	-	-	-	6, 5-OH
6	104.13	d	6.690	1	d	2.0	8, 5-OH
7	155.83	s	-	-	-	-	6, 8, 5-OH
8	101.37	d	7.101	1	d	2.0	6
8a	154.87	s	-	-	-	-	8
9	121.68	s	-	-	-	-	13
10	115.51	d	7.752	1	d	2.2	13, 14, 11-OH
11	145.15	s	-	-	-	-	13, 10*, 11-OH*, 12-OH*
12	148.21	s	-	-	-	-	10, 13, 14, 11-OH*, 12-OH*
13	115.67	d	6.904	1	d	8.5	12-OH, 14*, 10*
14	120.33	d	7.597	1	dd	2.2, 8.5	10, 13
15	163.88	s	-	-	-	-	*o*
ipso	117.71	s	-	-	-	-	*o*
ortho	109.39	d		2	s	-	*o*, *m*-OH
meta	145.85	s	-	-	-	-	*o*, *m*-OH
para	139.72	s	-	-	-	-	*o*, *m*-OH
3-OH	-	-	9.647	1	s	-	-
5-OH	-	-	12.593	1	s	-	-
11-OH	-	-	9.293	1	s	-	-
12-OH	-	-	9.660	1	s	-	-
m-OH	-	-	9.424	1	s	-	-
p-OH	-	-	9.195	1	s	-	-

* weak correlation.

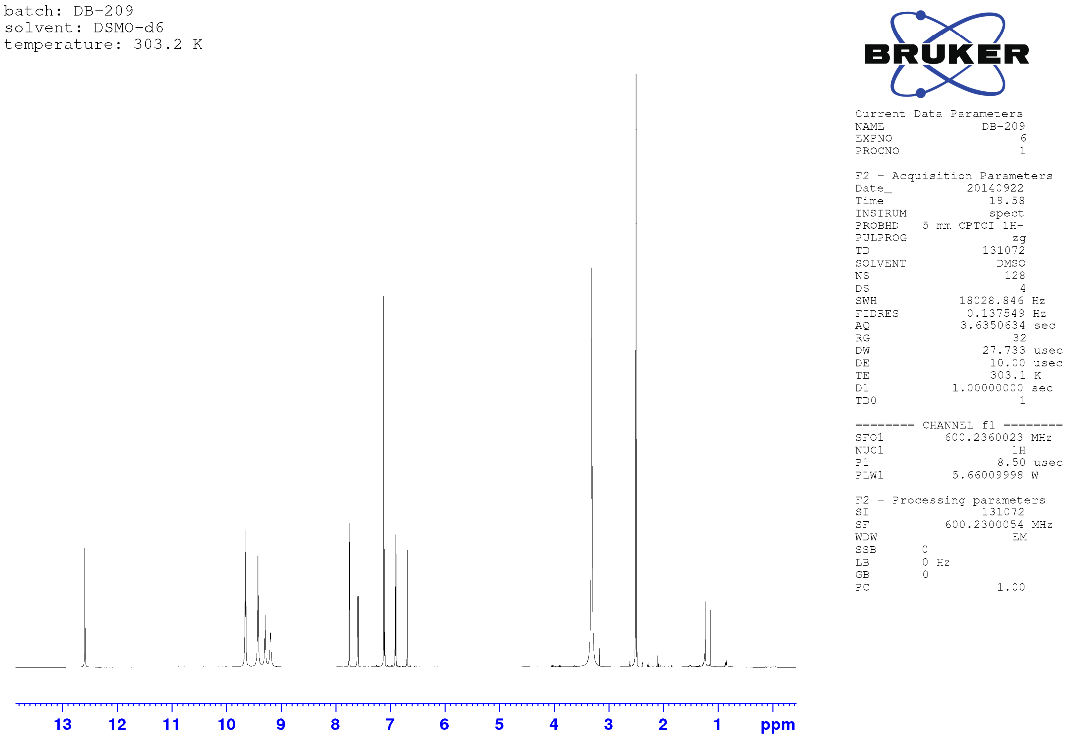

Figure S2. ^{1}H NMR spectrum of 7-*O*-galloylquercetin (**3**).

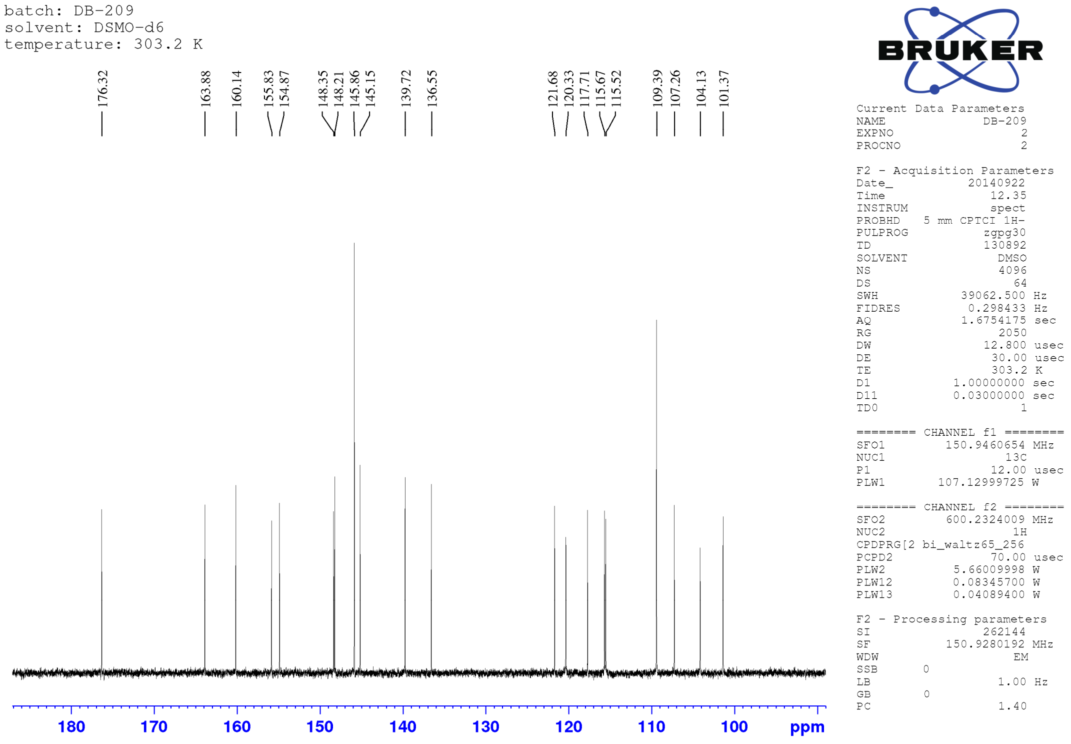

Figure S3. ^{13}C spectrum of 7-*O*-galloylquercetin (**3**).

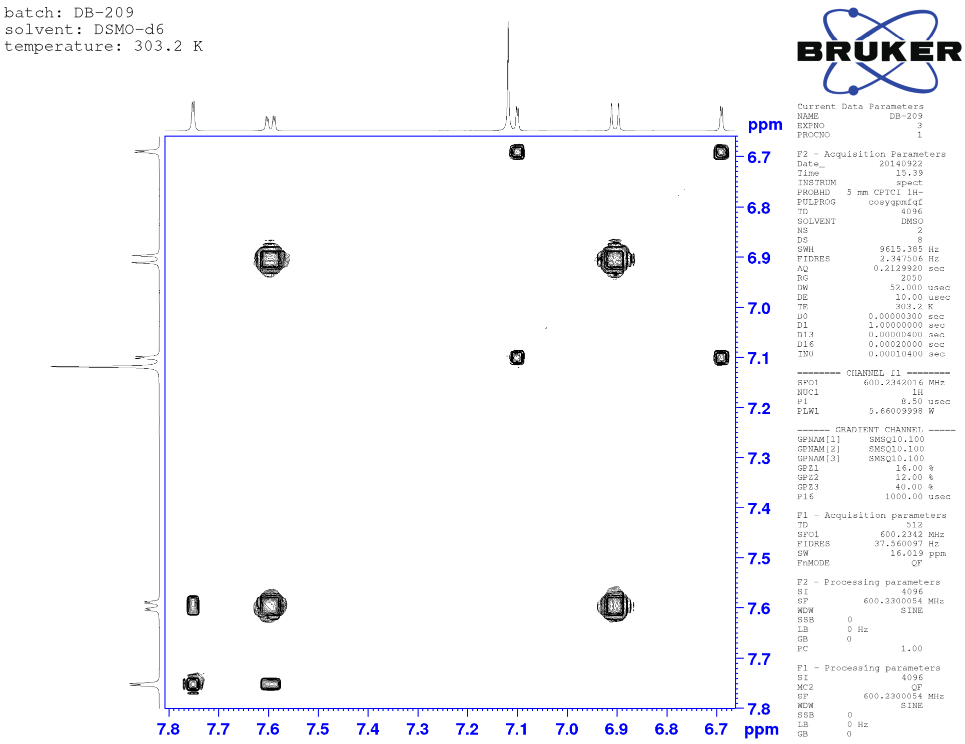

Figure S4. COSY spectrum of 7-*O*-galloylquercetin (**3**).

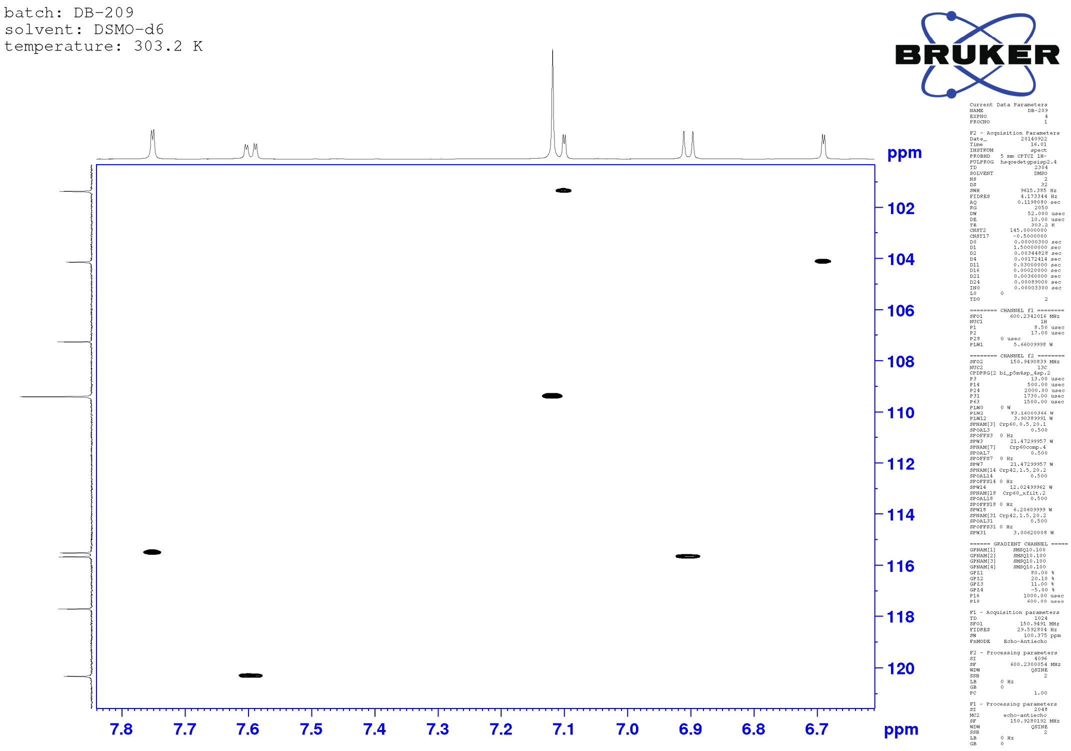

Figure S5. HSQC spectrum of 7-*O*-galloylquercetin (**3**).

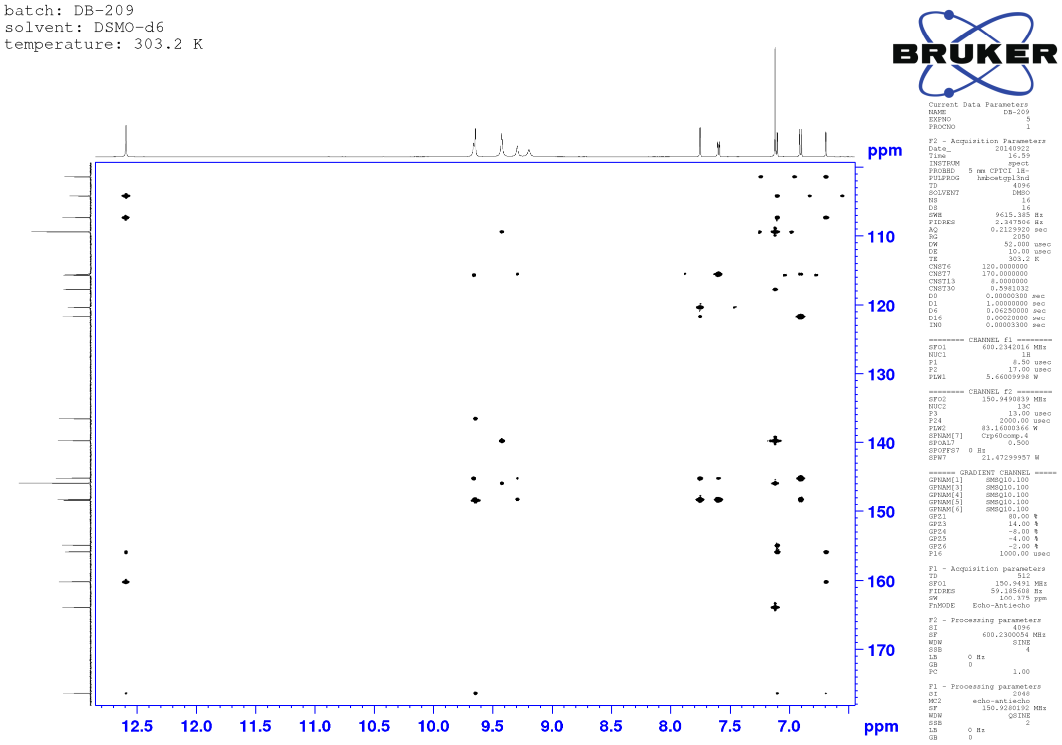

Figure S6. HMBC spectrum of 7-*O*-galloylquercetin (**3**).

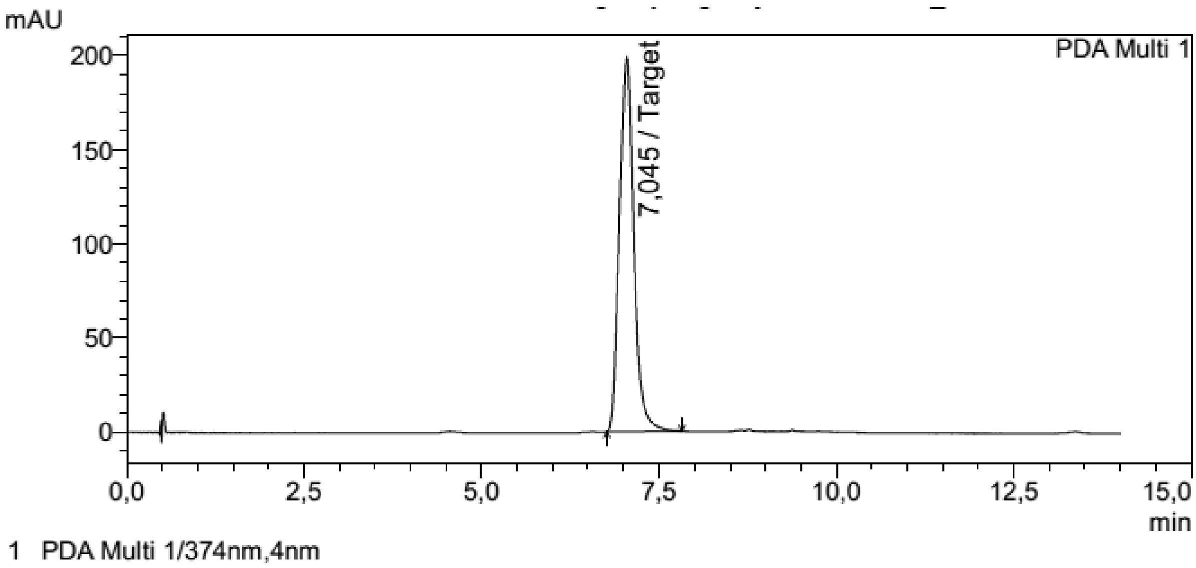

Figure S7. HPLC chromatogram of 7-*O*-galloylquercetin (**3**).

Method: Acetonitrile/water/formic acid (5/95/0.1, *v*/*v*/*v*, phase A) and water/formic acid (100/0.1, *v*/*v*, phase B); gradient: 0–4 min 15–25 % A, 4–7 min 25 % A, 7–9 min 25–60 % A, 9–12 min 60 % A, 12–12.5 min 60–15 % A; flow rate 1.2 mL/min at 25 °C. The PDA data were acquired in the 200–450 nm range and the 374 nm signal was extracted.

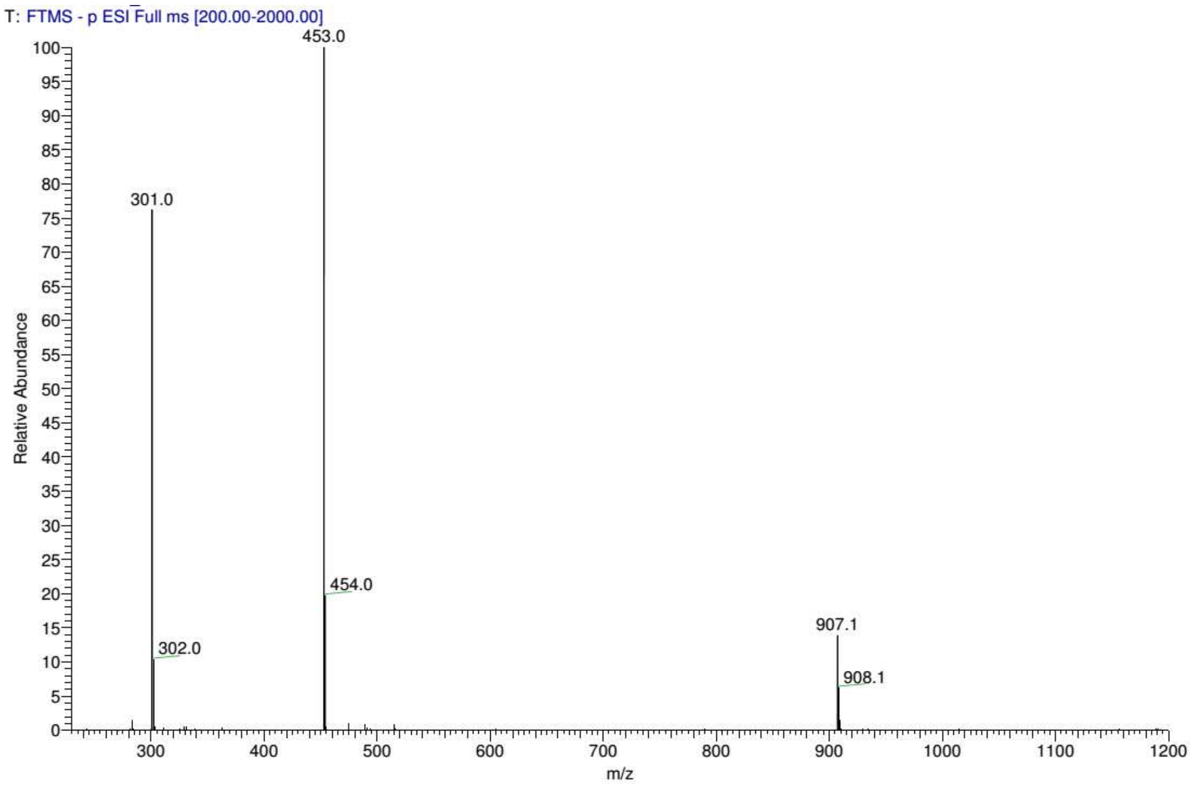

Figure S8. MS data of 7-*O*-galloylquercetin (**3**) (negative ESI).

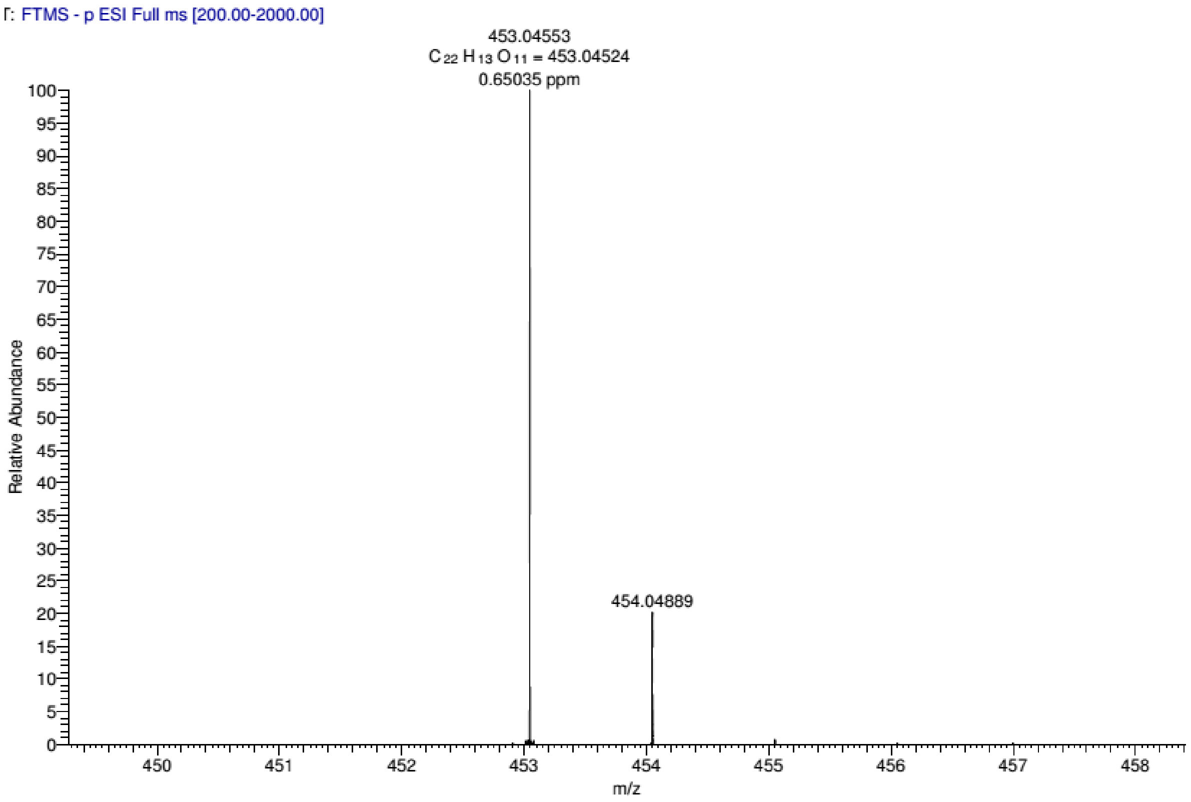

Figure S9. HRMS data of 7-*O*-galloylquercetin (**3**) (negative ESI).
